# Changes in salivary physiological stress markers induced by muscle stretching in patients with irritable bowel syndrome

**DOI:** 10.1186/1751-0759-2-20

**Published:** 2008-11-04

**Authors:** Toyohiro Hamaguchi, Shin Fukudo, Motoyori Kanazawa, Tadaaki Tomiie, Kunihiko Shimizu, Mineo Oyama, Kohji Sakurai

**Affiliations:** 1 Department of Occupational Therapy, School of Health and Social Services, Saitama Prefectural University, Saitama, Japan; 2 Department of Behavioral Medicine, Tohoku University, Graduate School of Medicine, Sendai, Japan; 3 Department of Occupational Therapy, Niigata University of Health and Welfare, Graduate School of Health Science, Niigata, Japan

## Abstract

**Background:**

Psychophysiological processing has been reported to play a crucial role in irritable bowel syndrome (IBS) but there has been no report on modulation of the stress marker chromogranin A (CgA) resulting from muscle stretching. We hypothesized that abdominal muscle stretching as a passive operation would have a beneficial effect on a biochemical index of the activity of the sympathetic/adrenomedullary system (salivary CgA) and anxiety.

**Methods:**

Fifteen control and eighteen untreated IBS subjects underwent experimental abdominal muscle stretching for 4 min. Subjects relaxed in a supine position with their knees fully flexed while their pelvic and trunk rotation was passively and slowly moved from 0 degrees of abdominal rotation to about 90 degrees or the point where the subject reported feeling discomfort.

Changes in the Gastrointestinal Symptoms Rating Scale (GSRS), State Trait Anxiety Inventory (STAI), Self-rating Depression Scale (SDS), ordinate scale and salivary CgA levels were compared between controls and IBS subjects before and after stretching. A three-factor analysis of variance (ANOVA) with period (before vs. after) as the within-subject factor and group (IBS vs. Control), and sex (men vs. female) as the between-subject factors was carried out on salivary CgA.

**Results:**

CgA showed significant interactions between period and groups (F[1, 31] = 4.89, p = 0.03), and between groups and sex (F[1, 31] = 4.73, p = 0.03). Interactions between period and sex of CgA secretion were not shown (F[1, 3] = 2.60, p = 0.12). At the baseline, salivary CgA in IBS subjects (36.7 ± 5.9 pmol/mg) was significantly higher than in controls (19.9 ± 5.5 pmol/mg, p < 0.05). After the stretching, salivary CgA significantly decreased in the IBS group (25.5 ± 4.5 pmol/mg), and this value did not differ from that in controls (18.6 ± 3.9 pmol/mg).

**Conclusion:**

Our results suggest the possibility of improving IBS pathophysiology by passive abdominal muscle stretching as indicated by CgA, a biochemical index of the activity of the sympathetic/adrenomedullary system.

## Background

Paying attention to the gut may magnify perceptions of abdominal symptoms and symptom related emotion [1,2]. Irritable bowel syndrome (IBS) is associated with an increased incidence of psychological disorder in patient populations [3], and while the cause and nature of this association are a matter of discussion, several possible mechanisms, both psychological and physiological, have been proposed to account for the finding [4-6]. Although there are many treatment strategies [7], traditional IBS therapy is mainly symptom oriented and often unsatisfactory. Increasing knowledge of brain-gut physiology [8], mechanisms, and neurotransmitters and receptors [9] involved in gastrointestinal motor and sensory function have led to the development of several new therapeutic approaches [10,11].

No single medication has proven to be universally effective, and the multiple therapeutic approach of gastrointestinal neurophysiology has led to promising advances in medical and non-medical approaches to IBS. Most studies have examined the association between mood state and IBS symptom severity using between-subjects design. The mechanisms involved suggest an association between mood state and IBS symptom severity within the individual. For example, although self-report measures of symptom severity cannot distinguish between the effect of mood state on physiology and on symptom perception, both mechanisms would lead to a situation in which a worsening of mood would occur before a worsening of IBS symptoms when both are measured longitudinally.

Autonomic imbalance has been proposed as a pathophysiological factor of IBS. Adrenergic neural activity and rectal sensitivity are more pronounced in IBS patients than in normal controls [12]. The stress response system includes the sympathetic/adrenomedullary (S/A) system and the hypothalamic-pituitary-adrenal (HPA) axis. The activities of the HPA axis and the S/A system can be biochemically evaluated by measuring catecholamines and cortisol, and we can measure these hormones as objective markers of stress. Recently, as a result of investigating the derivatives of catecholamines that are detectable in saliva, chromogranin A (CgA) was determined to be a useful index of psychological stress. CgA is a member of a family of highly acidic proteins, chromogranins, which are co-stored in the adrenergic neurons and paraneurons and co-released with adrenaline and noradrenaline in response to stimulation [13,14]. The changes in salivary CgA secretion resulting from exposure to a cognitive task may indicate psychological stress in humans [15].

Colonic stimulation results in brain activation of the somatosensory, insular, anterior cingulate and prefrontal cortices [2]. The somatosensory cortex receives direct anatomical projections from the ventral posterior thalamic nucleus, it is generally assumed that the somatosensory cortex is involved in parallel processing of tactile sensory information derived from this thalamic source of input [16]. In contrast, psychological stress influences pain thresholds via activation of the prefrontal cortices. Corticotropin releasing hormone is released from the hypothalamus, binding to visceral muscles and causing abnormal movement of the colon [9,17]. A stress marker of the S/A system, CgA, is released in saliva due to negative feelings such as aversive stimuli and psychological stress [15,18]. Mental activity may modulate gut perception [18,19] and override the effect of somatic stimulation on gut perception. For example, afferent signals from muscle stretching might modulate visceral perception and emotion via the spinothalamic pathway.

Skeletal muscle stretching is a unique method for relaxation [20-22]. The effect of hypnotherapy on IBS has been well documented [23], but specific psychotherapy usually needs long-range training for therapists at much cost. On the other hand, skeletal muscle stretching is simple and applicable in daily practice. Skeletal muscle stretching improved subjective pain scores of the patients with low back pain, and salivary cortisol concentrations were also significantly decreased during exercise [24]. However, the effects of skeletal muscle stretching on IBS are still unknown.

We hypothesized that IBS subjects would show abnormal salivary CgA and that skeletal muscle stretching would have beneficial effects on the pathophysiology of IBS.

## Methods

### Subjects

This study was approved by the Ethics Committee of Niigata University of Health and Welfare. All subjects gave their written informed consent. The subjects were university students, including 15 healthy volunteers as controls (7 males, 8 females: university students at Niigata University of Health and Welfare) and 18 subjects with IBS (not receiving medical treatment for IBS, 7 males, 11 females) aged 20 to 23 years old. The IBS subjects were 20 subjects selected from 245 volunteers selected through a pre-designed questionnaire based on the Rome III criteria [25] for functional gastrointestinal diseases. Two IBS subjects were excluded from the results because of incomplete examinations due to cold and headache.

As recommended by the Rome III committees [25], patients with IBS were classified by the predominant stool pattern: IBS with diarrhea (IBS-D) was defined as loose (mushy) or watery stool >25% and hard or lumpy stool <25% of bowel movements; and IBS with constipation (IBS-C) as hard or lumpy stool 25% and loose or watery stool <25% of bowel movements. Based on questions about the proportion of bowel movements that were either loose or watery, or hard or like a ball (lumpy), IBS subjects were classified by Rome III criteria [25] as IBS-D (n = 4), IBS-C (n = 8), IBS-M (n = 2) and IBS-U (n = 4).

### Stretching of the abdominal muscles

Subjects attended a preliminary test session that included the measurement of psychological characteristics and maximal abdominal muscle stretch. Subjects relaxed in a supine position with their knees fully flexed while their pelvis and trunk were passively and slowly moved from 0 degrees of abdominal rotation to about 90 degrees or until the subject reported feeling discomfort.

Participants attended one of several 30-minute experimental stretch sessions that were conducted at the same time of day. Subjects were instructed not to begin a stretching program session and to reschedule their session, if symptoms of their IBS prevented the stretching. During the session, subjects engaged in a 1-minute cyclic stretching protocol, 2 times right and left side rotation of their pelvis and trunk, and a 4-minute static stretching protocol. For the 4-minute static stretching protocol, the subject's knees were moved at a rate of 30°/s from 90 degrees of trunk and pelvic rotation (neutral) to a static hold at 80% of the subject's maximal passive rotation angle for one minute [26]. Immediately following the static stretching, the knees were returned to neutral, then moved to 80% of maximal angle on the other side, and again returned back to neutral [27]. The last stretching sequence was necessary so that measurements of stiffness and abdominal discomfort before the stretching could be compared with measurements of sensation and emotion after the stretching.

### Measurement of symptoms and psychological status

Before the experiment, gastrointestinal (GI) symptoms and psychological status were evaluated using the Gastrointestinal Symptoms Rating Scale (GSRS) [28], Zung's Self-rating Depression Scale (SDS) [29], and the State-Trait Anxiety Inventory (STAI) [30]. In addition, the subjects were asked to report the following seven items of visceral sensation or emotion [2]: abdominal discomfort, abdominal distention, abdominal pain, urgency for defecation, perceived stress, sleepiness, and anxiety before and after perceived stretching. Each sensation was evaluated on a scale from 0 (no sensation) to 10 (maximal sensation) as previously described [2,31].

GSRS is a 15-item instrument designed to assess the symptoms associated with common GI disorders. It has five subscales (Reflux, Diarrhea, Constipation, Abdominal Pain, and Indigestion Syndrome). Subscale scores range from 1 to 7 and higher scores represent more discomfort. A total score is derived by summing the individual item scores, and ranges from 15 to 105 [32].

SDS is a 20-item self-report questionnaire. Each item is scored on a Likert scale ranging from 1 to 4. A total score ranges from 20 to 80. Most people with depression score between 50 and 69, while a score of 70 and above indicates severe depression [33].

Mean scores of STAI for normal subjects were substantially lower than those reported in the English STAI Manual (State 24.95 ± 11.36 vs. 36.54 ± 10.22 and the Trait score was 27.88 ± 11.43 vs. 35.55 ± 9.76). The reported scores for depressed patients were 56.22 ± 8.86 and 53.83 ± 10.87, The state score for healthy subjects was 34.30 ± 10.79 and the Trait score was 36.07 ± 10.47 [34].

### Salivary CgA sampling

Salivary samples were collected immediately before and after stretching. Saliva samples were extracted from cotton wads that subjects held in their mouths for 2 min by centrifuging at 3,000 rpm for 15 min. The tactile stimulation of the presence of the cotton wad in the oral cavity tends to stimulate a rather uniform salivary flow [35]. During collection, the cotton wad was rolled around like a hard candy in the oral cavity. The samples were stored at -20°C until the assay. Salivary CgA levels were determined using an enzyme-linked immunosorbent assay (EIA) kit (YK070 Human Chromogranin A EIA, Yanaihara Institute, Inc., Shizuoka, Japan), using the method of Yanaihara et al. [36] of Yanaihara Laboratories (Fujinomiya, Shizuoka, Japan). The corrected values of CgA (pmol/mg) were calculated by dividing by the raw results of EIA with the protein concentration of the saliva in the samples (pg/mg).

Levels of salivary CgA were evaluated, according to a previously described method [35,37,38]. Salivary CgA levels are reported as being within the range of 50.0 ± 40.0 pmol/mg (protein corrected) in healthy subjects [39]. Salivary CgA might be a sensitive and promising index for psychosomatic stress. Therefore, an understanding of the circadian rhythm of salivary CgA in normal humans is important. According to a recent report, CgA does not show any obvious circadian rhythm. Salivary CgA levels peak upon awakening, and then quickly decrease to the nadir after 1 hour and maintain a low level throughout the day [40]. The circadian variation of CgA is still not fully established.

### Statistic analysis

A three-factor analysis of variance (ANOVA) with period (before vs. after) as the within-subject factor and group (IBS vs. Normal) and sex (men vs. female) as the between-subject factors, was carried out on salivary CgA. Changes in revised salivary CgA levels between before and after stretching sessions for each group were analyzed statistically by related-measures 3-factor ANOVA, followed by Bonferroni protected least significant difference for multiple comparisons; values of p < 0.05 were considered significant.

Values of visceral perception and emotion were compared between groups with the Mann-Whitney U test. Spearman's rank correlation was used for evaluating the intragroup correlation coefficient between CgA and psychological status and perceptional/emotional ratings; values of p < 0.05 were accepted as significant.

## Results

### GI symptoms and psychological status

Table 1 shows the scores for GI symptoms and psychological status in the Normal and IBS groups. The GSRS score of IBS subjects was significantly higher than that of Normal subjects (Mann-Whitney's U test, p = 0.01). State anxiety and trait anxiety scores of STAI of IBS subjects were significantly higher than those of normal subjects (p < 0.01). SDS scores did not significantly differ between Normal and IBS subjects.

**Table 1:** Comparisons of GI symptoms and psychological status between IBS subjects and normal controls

	Controls (n = 15)	IBS (n = 18)	p
GSRS	32.47 ± 5.21	51.16 ± 4.95*	<0.01
STAI-S	23.20 ± 3.23	41.90 ± 1.62*	<0.01
STAI-T	25.53 ± 3.22	42.53 ± 1.80*	<0.01
SDS	42.00 ± 1.60	45.21 ± 1.73	0.23

Mean ± Standard Error. *Comparison between normal group vs. IBS group by the Mann-Whitney's U test. GSRS: Gastrointestinal Symptoms Rating Scale, STAI-S: state anxiety, STAI-T: trait anxiety, SDS: Self-rating Depression Scale.

Abdominal discomfort in IBS subjects before and after stretching was significantly higher than in Normal subjects (p = 0.03) (Table 2). After stretching, there was no difference in abdominal discomfort between the groups. Anxiety was significantly decreased by stretching in Normal subjects (p = 0.04).

**Table 2:** Ratings of perception and emotion

Perception and emotion	Normal (n = 15)		IBS (n = 18)	
	
	Before	After	Before	After
Abdominal discomfort	0.27 ± 0.12	0.20 ± 0.11	1.84 ± 0.43*^a^	1.74 ± 0.37*^b^
Abdominal distention	0.40 ± 0.16	0.40 ± 0.16	1.32 ± 0.39	1.05 ± 0.35
Abdominal pain	0.20 ± 0.11	0.27 ± 0.15	0.74 ± 0.30	0.47 ± 0.25
Urgency	0.33 ± 0.16	0.47 ± 0.19	0.32 ± 0.23	0.26 ± 0.19
Stress	2.40 ± 0.54	2.27 ± 0.57	2.21 ± 0.41	2.37 ± 0.50
Sleepiness	3.00 ± 0.39	3.53 ± 0.45	4.42 ± 0.72	5.00 ± 0.64
Anxiety	2.20 ± 0.48	0.87 ± 0.35*^c^	2.42 ± 0.47	1.90 ± 0.48

Mean ± Standard Error. *a: Comparison between Normal group vs. IBS group before stretching (p = 0.03), *b: Normal group vs. IBS group after stretching (p = 0.03), *c: before vs. after stretching of the Normal group (p = 0.04) by Mann-Whitney's U test.

Two-way ANOVA of abdominal discomfort showed that there were significant period × group interactions (F [3, 62] = 6.82, p = 0.005). Before stretching, abdominal discomfort in IBS subjects (1.84 ± 0.43) was significantly higher than in Normal subjects (0.27 ± 0.12, post-hoc, p = 0.02). After stretching, abdominal discomfort in IBS subjects (1.74 ± 0.37) was significantly higher than in Normal subjects (0.20 ± 0.11, post-hoc, p = 0.01). Abdominal discomfort changes between before and after stretching in Normal controls were not significant (post-hoc, p = 0.89). There were no changes in the other scales before and after stretching or between normal controls and IBS subjects.

### Changes of salivary CgA

**Figure 1:**
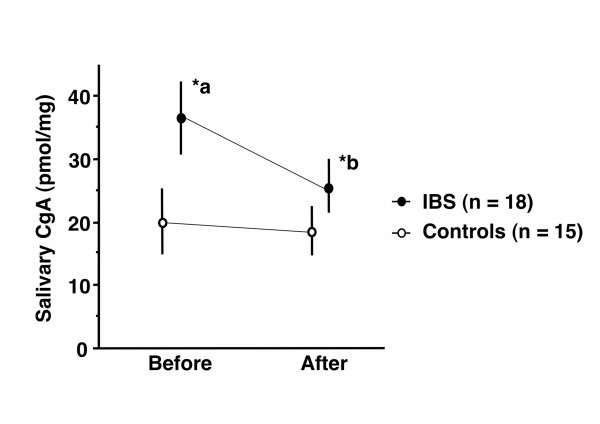
**Three-way repeated-measures ANOVA of CgA showed significant interactions between period and groups (F[1, 31] = 4.89, p = 0.03)**. *a: Before stretching, salivary CgA in IBS subjects (36.7 ± 5.9 pmol/mg) were significantly higher than in Normal controls (19.9 ± 5.5 pmol/mg, post-hoc, p = 0.006). A significant CgA change between before and after stretching in Normal controls was not shown (post-hoc, p = 0.60). *b: CgA was significantly decreased after stretching in IBS subjects (22.5 ± 4.5 pmol/mg, post-hoc, p = 0.02). After stretching, there was no significant difference in CgA between Normal controls and IBS subjects (post-hoc, p = 0.22). Error bars are mean ± standard error.

For multiple group comparisons, homogeneity of variance was assessed by the Levene test. Three-way repeated-measures ANOVA of CgA showed significant interactions between period and groups (F[1, 31] = 4.89, p = 0.03), and between groups and sex (F[1, 31] = 4.73, p = 0.03). Interactions between period and sex of CgA secretion were not significant (F[1, 3] 2.60, p = 0.12). Before stretching, salivary CgA in IBS subjects (36.7 ± 5.9 pmol/mg) was significantly higher than in Normal subjects (19.9 ± 5.5 pmol/mg, post-hoc, p = 0.006) (Fig. 1). CgA changes before and after stretching in Normal subjects were not significant (post-hoc, p = 0.60). In contrast, CgA was significantly decreased after stretching in IBS subjects (22.5 ± 4.5 pmol/mg, post-hoc, p = 0.02). After stretching, there was no significant difference in CgA between Normal and IBS subjects (post-hoc, p = 0.22).

The Spearman rank correlation coefficient showed a significantly positive correlation between CgA secretion before stretching and SDS score in IBS (r = 0.51, p = 0.03) (Table 3). The change in CgA after stretching compared with before stretching was positively correlated to the SDS score in both groups (IBS: r = 0.52, p = 0.03. Normal: r = 0.53, p = 0.04). In ratings of perception and emotion, perceived stress to stretching was negatively correlated with CgA secretion in the Normal group (r = -0.66, p = 0.007).

**Table 3:** Correlation between (r) CgA and GI symptoms and psychological status before and after stretching for the Normal and IBS groups.

	GSRS	SDS	STAI-S	STAI-T	Abdominal Discomfort	Abdominal Pain	Stress	Anxiety
**IBS **(n = 18)								
before	-0.16	0.51*	-0.17	-0.30	0.11	-0.13	-0.20	0.05
after	-0.34	0.19	-0.41	-0.29	0.24	-0.20	-0.37	-0.21
ΔCgA	0.08	0.52*	0.04	0.09	0.10	-0.05	0.18	0.36
**Normal **(n = 15)								
before	0.07	0.24	0.16	0.44	-0.23	0.13	-0.53*	-0.01
after	-0.20	0.08	0.15	0.07	-0.36	-0.04	0.09	0.18
ΔCgA	0.26	0.53*	-0.01	0.40	0.09	0.25	-0.66*	0.04

Significance, *p < 0.05. ΔCgA: CgA secretion before stretching – CgA after stretching.

## Discussion

This is the first study to demonstrate that the salivary CgA level of IBS subjects is higher than that of normal subjects. We also demonstrated that, after stretching, the CgA level of IBS subjects became comparable with that of normal subjects. CgA is a major soluble protein in adrenal medullary chromaffin granules and adrenergic neurons and is co-released with catecholamines, which are considered to be a good index of sympathetic activity [41,42]. In particular, salivary CgA is reported to be a sensitive and substantial marker of psychological stress, which does not respond well to physical stress [15]. The results of this study suggest that abdominal muscle stretching may improve sympathetic arousal in IBS subjects.

Patients with IBS show more psychiatric disorders and pathologic behavioral patterns than normal subjects [4,43]. A correlation between CgA secretion and the depression score was observed in this study. This result suggests a potential mechanism connecting events in the nervous system (central or enteric) with IBS symptoms. Noradrenaline in the brain plays a crucial role in anxiety, and colorectal distention induces noradrenaline release in the hippocampus [44]. Not only central but also peripheral adrenergic/noradrenergic functions may contribute to the pathophysiology of IBS. Elsenbruch et al. reported that IBS patients demonstrated significantly greater postprandial increases in plasma noradrenaline and systolic blood pressure [45]. In inflammatory bowel diseases, disturbed adrenergic regulation of interleukin-10 (IL-10) could be part of the mechanism underlying the modulation of disease activity due to psychological stress [46]. Disturbed autonomic or neuroendocrine modulation of cytokine production, may play a role in the pathogenesis of IBS [9]. Increased salivary CgA in IBS subjects suggests that IBS subjects have sympathetic arousal due to increased signaling to the gut afferent neurons.

IBS symptoms are generally worsened by stress and often improve with physical exercise and medications affecting serotonin function [47-49]. Sugano et al. reported that the skeletal muscle stretching program improved subjective pain scores of the patients with low back pain and that salivary cortisol concentrations were also significantly decreased up to 90 min after exercise [24]. Exercise may have beneficial effects on IBS symptoms [50].

CgA secretion before stretching was negatively correlated with the stress score of normal subjects. Additionally, the anxiety score was reduced after stretching in the normal subjects. Psychological factors influencing symptom reporting have been identified in the constructs of visceral perceptional amplification and alexithymia [51]. From a psychological viewpoint, IBS may be conceived as an abnormal cognitive processing of emotional stimuli, via verbal responses, and a tendency to perceive somatic stimuli as evidence of symptoms of disease.

Ghoncheh et al. examined the psychological and physical effect of passive muscle stretching and yoga stretching exercises for relaxation [22]. Muscle relaxation displayed higher levels of relaxation states, physical relaxation, disengagement and higher levels of joy as a post-training effect [52]. Muscle stretching provides sensation contrasts for learning relaxation in addition to fostering relaxation through the stretching of muscles [53]. Muscle stretching for patients with IBS may be of benefit to the patients and could be used as part of a multi-component approach to the treatment of IBS.

Evidence of a physiological component of IBS is based on gender differences in GI symptoms, central nervous system pain processing, and specific effects of estrogen and progesterone on gut function [54,55]. Additional factors may play a role, including gender-related differences in neuroendocrine, S/A system, and stress reactivity, which are related to bowel function and pain. Although gender differences in the therapeutic benefits of serotonergic agents have been observed [56], less is known about potential differences in responsiveness to non-drug therapies for IBS. Multiple comparisons between CgA and gender related information suggest that stretch intervention may have gender dependent effects on IBS.

The following three points can be cited as limitations of this study. The first is that sample size was very small. The levels of CgA found in our subjects were somewhat different from the reported mean value [39], and our findings could not exclude the effect of the sample size. Additionally, we could not examine the effects on subtypes of IBS (i.e. constipation-predominant or diarrhea-predominant), because the sample sizes of the subtypes were too small to analyze them separately. However, a long follow-up study [57] proved the inconsistency of IBS subtypes, suggesting that whole IBS analysis is more important than subtype analysis. The second limitation is that the duration of muscle stretching might be too short. The duration of the effect of contraction-relaxation stretching on range of motion (ROM) in the lower extremities is 15 min and the increase in ROM usually remains for 90 min [58]. Proprioceptive neuromuscular facilitation (PNF) stretching techniques produced greater increases in ROM than static or dynamic stretching exercises. The stretching hold time at the hip is 3–10 sec in one hold-relax PNF stretch [59]. There is no study that clarifies the stretch duration required of the abdominal muscle for relaxation. Thus, it will be necessary to examine how long we should stretch the abdominal muscles for IBS treatment. Lastly, we could not analyze the effect of lifestyle and medical history on CgA in this study. Many stress-related biomarkers are affected by lifestyle or medical history [60]. Such relationships might contribute to increased knowledge about strategies to prevent progression of IBS.

## Conclusion

Our results suggested that it is possible to improve IBS pathophysiology by passive abdominal muscle stretching using a biochemical index of the activity of the S/A system (salivary CgA). In this study, we verified only the effects of stretching and presence of IBS on CgA levels. Further study of the S/A system and muscle stretching in IBS is warranted.

## Abbreviations

IBS: irritable bowel syndrome; CgA: chromogranin A; GSRS: Gastrointestinal Symptoms Rating Scale; STAI: State Trait AnxietyInventory; SDS: Self-rating Depression Scale; ANOVA: analysis ofvariance; S/A: sympathetic/adrenomedullary.

## Competing interests

The authors declare that they have no competing interests.

## Authors' contributions

TH was the main investigator and wrote the first draft of the manuscript. SF supervised the study, analyzed the data and wrote the final draft of the manuscript. MT and TT supervised the study. KS, MO and KS contributed to the study design. KS contributed to the data collection. All authors contributed to the preparation of the article and approved the final manuscript.
